# Increased insulin sensitivity in individuals with neurofibromatosis type 1

**DOI:** 10.20945/2359-3997000000007

**Published:** 2018-01-01

**Authors:** Aline Stangherlin Martins, Ann Kristine Jansen, Luiz Oswaldo Carneiro Rodrigues, Camila Maria Matos, Marcio Leandro Ribeiro Souza, Débora Marques Miranda, Nilton Alves de Rezende

**Affiliations:** 1 Universidade Federal de Minas Gerais Universidade Federal de Minas Gerais Centro de Referência de Neurofibromatose Belo Horizonte MG Brasil Centro de Referência de Neurofibromatose, Universidade Federal de Minas Gerais (UFMG), Belo Horizonte, MG, Brasil; 2 Universidade Federal de Minas Gerais Universidade Federal de Minas Gerais Departamento de Nutrição Belo Horizonte MG Brasil Departamento de Nutrição, Universidade Federal de Minas Gerais (UFMG), Belo Horizonte, MG, Brasil; 3 Universidade Federal de Minas Gerais Universidade Federal de Minas Gerais Departamento de Clínica Médica Belo Horizonte MG Brasil Departamento de Clínica Médica, Universidade Federal de Minas Gerais (UFMG), Belo Horizonte, MG, Brasil

**Keywords:** Blood glucose, neurofibromatosis 1, diabetes mellitus, type 2, insulin resistance

## Abstract

**Objects:**

To compare insulin resistance (IR) and metabolic aspects of patients with neurofibromatosis type 1 (NF1) and individuals without the disease.

**Subjects and methods:**

Forty patients with NF1 were matched by sex, age, and body mass index (BMI) to 40 controls from the community. Blood samples were collected for biochemical assessment. Homeostasis model assessment adiponectin (HOMA-AD), Homeostasis model assessment insulin resistance (HOMA-IR), and adiponectin/leptin ratio (ALR) were used to identify IR.

**Results:**

The median HOMA-IR values were similar between the groups. However, the HOMA-AD value was significantly lower and the ALR significantly higher in the NF1 group. Fasting blood glucose (FBG), leptin, and visfatin levels of patients with NF1 were significantly lower, although adiponectin levels were significantly higher than those in the controls. Fasting insulin and blood glucose levels 2 hours after administration of 75 g of dextrose, glycated hemoglobin, and resistin showed no significant differences between groups. The HOMA-AD correlated with BMI, FBG, blood glucose levels 2 hours after administration of 75 g of dextrose, fasting insulin, glycated hemoglobin, adiponectin, leptin, visfatin, ALR, and HOMA-IR. The ALR correlated with BMI leptin, visfatin, and adiponectin.

**Conclusions:**

Lower levels of FBG, leptin, visfatin, and HOMA-AD, and higher adiponectin levels and ALR may be related to increased insulin sensitivity and lower occurrence of type 2 diabetes mellitus in patients with NF1

## INTRODUCTION

A rare disease is generally defined as a disease with a very low prevalence (1). Although considered individually rare, as a group, these diseases affect a significant percentage of the population and present a relevant health problem (2). Therefore, research is needed to better understand these diseases.

Neurofibromatosis type 1 (NF1) is a rare, autosomal-dominant disorder caused by inherited or new mutations on chromosome 17. These mutations result in dysfunction of the neurofibromin protein, which is involved in growth control and behavior of various tissues (3). It is one the most common human monogenic diseases, with an estimated prevalence of approximately 1: 3,500 births (4).

The clinical NF1 criteria include *café-au-lait* spots, neurofibromas, axillary and/or inguinal freckling, Lisch nodules in the iris, optical gliomas, specific bone dysplasia, and familial history (5). NF1 is a multisystem disease, affecting the musculoskeletal, cardiovascular, endocrine, and central and peripheral nervous systems as well as learning skills (6).

Data suggest a lower prevalence of type 2 diabetes mellitus (T2DM) in patients with NF1 (7,8). Madubata and cols. (9) showed that patients with NF1 had a lower chance of having DM compared to healthy individuals. Moreover, Martins and cols. (10) reported that patients with NF1 showed significantly lower fasting blood glucose (FBG) levels compared to controls without the disease.

T2DM (90% to 95% of all cases of diabetes) is associated with insulin resistance (IR) (11). The hyperinsulinemic euglycemic clamp technique is considered the gold standard to assess IR; however, it is a complex and expensive method that is often not available for studies and clinical practice (12). Another IR validated index is the homeostasis model assessment for insulin resistance (HOMA-IR) (13). This index shows a strong correlation with hyperinsulinemic euglycemic clamp findings (14). However, there is some debate regarding its ability to detect IR in individuals with normal glucose or impaired glucose tolerance (15).

Matsuhisa and cols. (16) developed a modified HOMA index, the HOMA-adiponectin (HOMA-AD), by including adiponectin levels in the denominator. This modification makes the HOMA index a more sensitive marker of IR (16,17).

Mediators produced by adipose tissue, called adipocytokines (leptin, visfatin, resistin, and adiponectin) affect glucose homeostasis, appetite regulation, inflammation, and atherosclerosis and are associated with IR (18). Studies have shown increased levels of leptin, visfatin, and resistin in individuals with T2DM (18). In contrast, adiponectin levels are reportedly lower in patients with DM and metabolic syndrome (19) and studies have shown that their levels may be markers of the risk of prediabetes (20).

In addition, some studies have suggested that the adiponectin-leptin ratio (ALR) may be an effective parameter for the assessment of IR in individuals with or without diabetes (21).

The results of previous studies lead us to hypothesize that patients with NF1 may have (1) genetic differences that modify glucose utilization and cellular control and/or (2) phenotypic characteristics that result in increased insulin sensitivity, favoring the maintenance of lower levels of FBG and reducing the risk of developing T2DM. Thus, this study aimed to evaluate IR and metabolic aspects of patients with NF1 compared with control individuals without the disease.

## MATERIALS AND METHODS

### Population

The study included patients over 20 years of age with NF1 followed at the Reference Center on Neurofibromatoses at the Federal University of Minas Gerais (CRNF-UFMG) who met at least three criteria for the diagnosis of disease according to the National Institute of Health (22).

The control group consisted of volunteers from the community (e.g., university students, CRNF-UFMG officials, and companions of patients seen at the clinic) who were similar in terms of sex, age, and BMI to the included patients with NF1.

Individuals diagnosed with diabetes, liver disease or malignancy, and infection as well as those using steroids, antibiotics, statins, insulin, and oral hypoglycemic agents were excluded from both groups.

### Sample

The sample size was calculated based on the results of the study by Martins and cols. (10), and we used a standard deviation of 13 units, a least significant difference of 10 units, and power of 90%, resulting in the requirement for a minimum of 37 participants in each group.

### Ethical aspects

The study was approved by the Ethics and Human Research of the UFMG (number 258.325, year 2013) and all participants signed an informed consent form.

### Procedures

Weight and height of the participants were measured, and BMI calculated based on protocols established by the World Health Organization (23).

We assessed the physical activity level by the International Physical Activity Questionnaire (24) and the diet by means of a 3-day non-consecutive dietary record. For each record, the total amounts of the following components were calculated using the food composition tables: calories, carbohydrates, lipids, proteins, fiber, cholesterol, saturated fatty acids, monounsaturated and polyunsaturated fatty acids, zinc, magnesium, selenium, and vitamin D.

Blood samples were collected from the participants for analysis of FBG, blood glucose 2 hours after administration of 75 g of dextrose, glycated hemoglobin (HbA1c), and fasting insulin, adiponectin, leptin, visfatin, and resistin according to the protocol used at the CRNF-UFMG.

Blood glucose and glycosylated hemoglobin (HbA1c) levels were measured using the Vitros equipment and reagents from Ortho-Clinical Diagnostics^®^ (blood glucose coefficient of variation [CV] inter-assay, level 1: 2.24% and level 2: 2.23%; HbA1c CV, level 1: 5.11% and level 2: 6.04%). Insulin levels were measured using the Architect-Abbott^®^ equipment, with Abbott^®^ reagents (CV inter-assay, level 1: 4.4, level 2: 2.9, and level 3: 3.2).

The leptin assay was performed using the human leptin enzyme-linked immunoassay (ELISA) kit from Millipore (CV inter-assay: 3.7%; CV intra-assay: 4.4%, respectively). Resistin levels were measured using the human resistin ELISA from eBioscience^®^ (CV interassay: 8.1%; CV intra-assay: 5.1%). Adiponectin levels were measured using the human adiponectin ELISA kit from eBioscience^®^ (CV inter-assay: 3.1%; CV intraassay: 4.2%, respectively). Visfatin was assayed using the human visfatin ELISA kit from MyBioSource^®^ (CV < 10%). The assays were performed according to the kit protocols.

FBG, HbA1c, and glucose levels 2 hours after administration of 75 g of dextrose were categorized according to the cutoff values established by the American Diabetes Association (11).

The markers of IR included the HOMA-IR (HOMAIR = fasting insulin (U/mL) × FBG (mmol/L)/22.5), HOMA-AD (HOMA-AD = fasting insulin (U/mL) × FBG (mmol/L)/Adiponectin (μ/mL) and ALR.

### Statistical analysis

Continuous variables were described as means and standard deviations or as medians and 25^th^ and 75^th^ percentiles as appropriate. Categorical variables were described as absolute and relative frequencies.

The Kolmogorov-Smirnov test was used to test the normality of the variables. Paired t-tests were used to compare groups with normally distributed variables. The Wilcoxon test was used to compare groups with non-normal variables distribution. McNemar's test was used to compare proportions between two groups. When McNemar's test could not be used, we used chisquare or Fisher's exact tests.

The Spearman correlation test was used to verify the correlations between HOMA-AD and ALR and other variables.

To determine whether the differences and associations were statistically significant, we used a 5% level of significance. Thus, P-values ≤ 0.05 were considered to represent statistically significant differences.

Data were analyzed using the Statistical Package for Social Science (SPSS) version 13.0.

## RESULTS

We evaluated 47 patients with NF1 and 47 controls. Seven individuals were excluded from the NF1 group (one because of the use of corticosteroids, one because of suspicion of malignancy, and five owing to unavailability of blood test results). The respective controls were also excluded, finally amounting to 40 participants in each group.

The general characteristics of patients with NF1 and controls are described in Table 1. The average weight and the median height were significantly lower in the NF1 group.

**Table 1:** General characteristics of patients with NF1 and controls

Variables		NF1 (n = 40)	Controls (n = 40)	P-value
Sex^a^				
	Female	28 (70.0)	28 (70.0)	1.000^d^
	Male	12 (30.0)	12 (30.0)	
Age^b^		40.7 (11.8)	40.4 (12.1)	0.390^e^
Weight (kg)^b^		60.6 (13.1)	68.5 (14.5)	< 0.001
Height (m)^c^		1.57 (1.51–1.63)	1.62 (1.57–1.74)	< 0.001
BMI (kg/m^2^)^c^		24.2 (21.6–26.2)	24.6 (22.3–26.5)	0.096^f^
Physical activity level*				
	Not active	9 (22.5)	14 (35)	
	Not very active	3 (7.5)	5 (12.5)	0.343^d^
	Moderately active	28 (70)	21 (52.5)	

a n (%);

b Mean (standard deviation);

c Median (percentiles 25-75);

d McNemar's test;

e Paired t-test;

f Wilcoxon Test; BMI: body mass index.

* According to the International Physical Activity Questionnaire (25).

There were no statistically significant differences between the NF1 and control groups regarding the intake of any of the evaluated nutrients.

Table 2 shows the IR markers and metabolic characteristics of patients with NF1 and controls. The HOMA-IR values were similar between groups. However, the HOMA-AD value was significantly lower and ALR was significantly higher in the NF1 group. The FBG, leptin, and visfatin levels in patients with NF1 were significantly lower than those in controls. The adiponectin levels was significantly higher in the NF1 group. No significant differences were observed between groups in the levels of resistin, post dextrose glucose, HbA1c, and fasting insulin.

**Table 2:** Insulin resistance markers and metabolic characteristics of patients with NF1 and controls

Variables		NF1 (n = 40)	Controls (n = 40)	P-value
HOMA-IR^a^		1.1 (0.7-1.6)	1.3 (0.9–2.3)	0.147^c^
HOMA-AD^a^		1.0 (0.5–1.7)	1.9 (1.0–4.1)	0.001^c^
ALR^a^		3.8 (2.1–12.6)	1.2 (0.7–2.1)	< 0.001^c^
FBG (mg/dL)^a^		83.5 (78.0–90.0)	86.0 (83.0–94.0	0.008^c^
FBG classification^b^				
	Normal (< 100 mg/dL)	40 (100.0)	35 (87.5)	0.027^d^
	Impaired (≥ 100 mg/dL)	0	5 (12.5)	
Glucose level postdextrose administration (mg/dL)^a^		96.5 (85.3–112.5)	99.5 (88.0–118.3)	0.255^c^
Glucose level postdextrose administration classification^b^				
	Normal (< 140 mg/dL)	38 (95.0)	34 (85.0)	0.219^e^
	Impaired (≥ 140 mg/dL)	2 (5.0)	6 (15.0)	
HbA1c (%)^a^		5.2 (5.0–5.5)	5.4 (5.0–5.7)	0.100^c^
HbA1c classification^b^				
	< 5.7%	30 (75.0)	26 (65.0)	
	5.7–6,4%	10 (25.0)	14 (35.0)	0.503^e^
	≥ 6.5%	0	0	
Fasting insulin (µU/mL)^a^		4.9 (3.6–11.6)	6.0 (4.4–15.7)	0.357^c^
Adiponectin (µg/mL)^a^		23.7 (17.0–39.4)	15.3 (11.3–20.9	0.001^c^
Leptin (ng/mL)^a^		5.7 (3.1–11.7)	11,9 (6.8–20.7)	0.042^c^
Resistin (ng/mL)^a^		6.8 (3.4–9.0)	5,8 (2.4–9.8)	0.490^c^
Visfatin (ng/mL)^a^		118.2 (105.8–124.8)	138.2 (125.5–147.7)	< 0.001^c^

a Median (percentiles 25 and 75);

b n (%);

c Wilcoxon test;

d Fisher's exact test;

e McNemar's test. HOMA-IR: Homeostasis Model Assessment Insulin Resistance; HOMA-AD: Homeostasis Model Assessment Adiponectin; FBG: fasting blood glucose; HbA1c: glycated hemoglobin; ALR: adiponectin-leptin ratio.

The HOMA-AD values were correlated with BMI, FBG, post dextrose glucose, fasting insulin HbA1c, adiponectin, leptin, and visfatin levels, as well as ALR and HOMA-IR values (Table 3).

**Table 3:** Variables significantly correlated with HOMA-AD values in all study participants (n = 80)

Variable	Correlation coefficient^a^	P-value
BMI	0.349	0.002
FBG	0.448	< 0.001
Fasting insulin	0.667	< 0.001
Post dextrose glucose	0.388	< 0.001
HbA1c	0.315	0.004
Adiponectin	-0.662	< 0.001
Leptin	0.300	0.007
Visfatin	0.269	0.016
ALR	-0.547	< 0.001
HOMA-IR	0.700	< 0.001

a Spearman correlation coefficient; FBG: fasting blood glucose; HbA1c: glycated hemoglobin; HOMA-IR: Homeostasis Model Assessment-Insulin Resistance; ALR: adiponectin-leptin ratio; HOMA-AD: Homeostasis Model Assessment-Adiponectin.

The ALR correlated with BMI (r = -0.239, p = 0.033), adiponectin (r = 0.652, p < 0.001), leptin (r = -0.869, p < 0.001) and visfatin levels (r = -0,329, p = 0,003).

## DISCUSSION

The results of the present study revealed that HOMA-IR was similar between the NF1 and control groups. However, the HOMA-AD values were significantly lower in patients with NF1 than those in the control group. FBG, leptin, and visfatin levels were significantly lower and adiponectin levels and ALR were significantly higher in patients with NF1 than those in the control group.

While the HOMA-IR is the most widely used index for the determination of IR, some authors have reported that this index fails to identify IR in individuals with normal glucose levels and impaired glucose tolerance (15). In this study, 100% of patients with NF1 showed FBG within normal levels and only 12.5% of controls had impaired FBG levels. Thus, the HOMA-IR may not be the most appropriate method for identifying IR in patients with NF1, in this sample.

HOMA-AD has been reported to be a suitable method for identifying IR in specific populations, and some authors consider it a more sensitive marker of IR (16,17). The results of this study revealed a significantly lower median HOMA-AD level in the NF1 group, suggesting that these patients may have greater insulin sensitivity than controls.

The ALR has also been reported to be a more efficient marker of IR than HOMA-IR, showing an inverse correlation with IR markers (21). In the present study, ALR was significantly higher in patients with NF1, also suggesting a greater sensitivity to insulin in this group of patients.

The mechanisms to explain increased insulin sensitivity in individuals with NF1 are unknown. It is known that excess adipose tissue plays an essential role in the development of IR. However, Pal and cols. (25) demonstrated that patients with mutations in the tumor suppressor phosphatase and tensin homologue, which causes a syndrome of predisposition to cancer, have increased insulin sensitivity and excess weight compared to controls. The authors have also shown that the excess weight found in the patients was not due to increased lean or bone mass, but rather to increased adiposity, and suggest that the excess weight may be due to the improved action of insulin in the adipose tissue.

Studies have shown that suppression of lipolysis is highly sensitive to insulin (26). This way, considering the conclusions of these studies, patients with NF1 who exhibit insulin sensitivity could be obese. However, our patients had BMI within the normal range, and other studies have already demonstrated a lower BMI in NF1 patients, contradicting the findings of Pal and cols. Two hypotheses could explain these findings, insulin sensitivity in individuals with NF1 may be mild, and it may not be sufficient to induce obesity or insulin sensitivity that is specific to non-fat tissues.

We believe that the characteristics of body composition in NFI patients, demonstrated in other studies (27) and associated with increased adiponectin and lower visfatin and leptin levels, may be related to higher insulin sensitivity found in this group of individuals.

Regarding metabolic characteristics, the median values of FBG in patients with NF1 were significantly lower than those in control individuals; the number of individuals with impaired FBG was also significantly lower in the NF1 group than in the control group. These data agree with a previous study by our group (10), which evaluated 57 patients with NF1 and 171 controls and found significantly lower levels of FBG in patients with NF1.

HbA1c, blood glucose 2 hours after administration of dextrosol, and fasting insulin levels were also similar between the groups, suggesting that some mechanism involved in the control of fasting glucose may be responsible for the lower levels of FBG in the NF1 group. After a prolonged period of fasting, glucose levels in the blood decline, stimulating the pancreatic alpha cells to release glucagon. The result is hepatic glycogenolysis and gluconeogenesis to increase blood glucose levels (28). Thus, we hypothesize that the lower levels of FBG in NF1 could be a result of a change in the glycogenolysis or gluconeogenesis process.

Neurofibromin plays a role in the regulation of the hypothalamus and pituitary gland (29), which are involved in the regulation of energy balance (30). Neurofibromin could play a role in glucose metabolism, and its deficiency may cause reduced gluconeogenesis in patients with NF1. Moreover, the modified pathways resulting in insulin changes related to neurofibromin are not clear.

A second hypothesis to explain the lower levels of FBG in patients with NF1 is derivate of the production of insulin-like growth factor 2 (IGF2) by neurofibromas. Studies have shown that tumors produce IGF2, which increases the consumption of peripheral glucose and decreases the glucose production in the liver, resulting in hypoglycemia (31). However, further studies are needed to verify if the FBG levels in patients with NF1 can be accounted for by IGF2 produced by neurofibromas.

A third hypothesis that could explain the lower fasting glucose levels in patients with NF1 is related to adipocytokine levels. The results of this study showed that adiponectin levels in patients with NF1 were significantly higher and levels of visfatin and leptin were significantly lower than in controls. According to Yamauchi and cols. (32) one of the mechanisms through which adiponectin may decrease the risk of T2DM is the suppression of hepatic gluconeogenesis. Leptin can reduce hepatic glucose production by decreasing the synthesis of phosphoenolpyruvate carboxylase, which is the key enzyme in gluconeogenesis (33). However, Gutiérrez-Juárez and cols. (34) showed that administration of leptin in rats stimulated gluconeogenesis. Visfatin, in turn, appears to stimulate gluconeogenesis (35).

Thus, differences in the levels of these adipocytokines may contribute to lower hepatic gluconeogenesis and lower levels of FBG in patients with NF1. However, further studies are needed to validate this hypothesis. The reason for the changes in the levels of these adipocytokines in patients with NF1 remains unknown. No other studies have evaluated adipocytokine levels in NF1.

The correlation of HOMA-AD values with diabetes mellitus markers, FBG, post dextrose glucose, and HbA1c in addition to HOMA-IR values, levels of adiponectin, leptin, and visfatin, and ALR reinforce the hypothesis that the characteristics observed in patients with NF1 (lower levels of FBG, visfatin, and leptin and higher adiponectin and adiponectin/leptin levels) may explain the lower incidence of T2DM in these patients.

This study has several limitations, including the small number of patients involved, although NF1 is a rare disease for which surveys are usually performed with even fewer participants. In addition, IR was not evaluated using the gold-standard techniques.

In conclusion, the results of this matched casecontrol study suggested for the first time that NF1 individuals have increased insulin sensitivity, as well as lower levels of FBG, visfatin, and leptin and higher adiponectin levels, than those without the disease. These characteristics may be associated with the lower occurrence of T2DM in this group of patients.
